# Testing a Gypsum Composite Based on Raw Gypsum with a Direct Admixture of Paraffin and Polymer to Improve Thermal Properties

**DOI:** 10.3390/ma14123241

**Published:** 2021-06-11

**Authors:** Krzysztof Powała, Andrzej Obraniak, Dariusz Heim

**Affiliations:** Faculty of Process and Environmental Engineering, Lodz University of Technology, 90-924 Lodz, Poland; andrzej.obraniak@p.lodz.pl (A.O.); dariusz.heim@p.lodz.pl (D.H.)

**Keywords:** phase-change material, gypsum, paraffin, thermal conductivity, paraffin tightness

## Abstract

The implemented new legal regulations regarding thermal comfort, the energy performance of residential buildings, and proecological requirements require the design of new building materials, the use of which will improve the thermal efficiency of newly built and renovated buildings. Therefore, many companies producing building materials strive to improve the properties of their products by reducing the weight of the materials, increasing their mechanical properties, and improving their insulating properties. Currently, there are solutions in phase-change materials (PCM) production technology, such as microencapsulation, but its application on a large scale is extremely costly. This paper presents a solution to the abovementioned problem through the creation and testing of a composite, i.e., a new mixture of gypsum, paraffin, and polymer, which can be used in the production of plasterboard. The presented solution uses a material (PCM) which improves the thermal properties of the composite by taking advantage of the phase-change phenomenon. The study analyzes the influence of polymer content in the total mass of a composite in relation to its thermal conductivity, volumetric heat capacity, and diffusivity. Based on the results contained in this article, the best solution appears to be a mixture with 0.1% polymer content. It is definitely visible in the tests which use drying, hardening time, and paraffin absorption. It differs slightly from the best result in the thermal conductivity test, while it is comparable in terms of volumetric heat capacity and differs slightly from the best result in the thermal diffusivity test.

## 1. Introduction

With the ever-growing industrial sector and the increasing trend of energy use, new technologies that reduce energy consumption are being developed. Due to the nature of electricity used and newer trends in energy saving—the latter of which is promoted in almost every area of society—there are many cases of uncontrolled loss of the effect of using energy, especially when it comes to heat energy [1]. Many people do not realize how much energy is lost due to poor-quality building materials, the way they are installed, or the failure to implement newly built materials that would improve many of the properties of basic building materials. Therefore, it is worth examining the new materials that can be added to existing and well-known materials, such as concrete or gypsum [2]. Many scientists are trying to develop the use of materials such as paraffin, fatty acids, and hydrated salts in relation to phase-change materials, which can themselves be used in a variety of applications [3,4,5,6].

There are many indications that PCM can be used in construction. Its benefits have been noted by scientists who study phase-change materials in most building layers, such as walls, ceilings, and floors [7,8,9,10]. One advantage of PCM as a phase-change material at a specific temperature is that it allows heat to be stored at the time of a phase change. It is interesting because, when changing the phase from solid to liquid, phase-change material achieves a high heat capacity, which translates into a greater amount of stored heat. An unquestionable advantage of PCM is its possible generation of such a large amount of heat because, after another phase change—this time from liquid to solid—the stored heat is released. An interesting fact about paraffin is that manufacturers can produce paraffin with different melting points, which means that it can be used wherever the average daily temperature is known, especially in relation to rooms where thermal comfort for people needs to be achieved. When there is a change in the state of aggregation into liquid PCM, heat energy is released, and then, e.g., at night, when the temperature is below melting point, the state of aggregation changes to a solid and the stored energy is released into the environment [11,12].

Phase-change materials have a characteristic way of storing energy in an almost isothermal manner. This is because energy is stored in the form of latent heat and is characterized by high storage capacity; it is also accompanied by slight temperature fluctuations from storage to release. In such a way, energy is stored during melting and recovered during solidification of the phase-change material. Compared to other methods of energy storage, such as sensible heat extraction or chemical storage, such a method is simple and easy to control, especially as phase-change material has a specific heat capacity depending on its type; this makes the process more predictable [13].

There are two main groups of phase-change material—organic and inorganic materials. There are also eutectic materials which, under certain melting point conditions, constitute the third group as a mixture of the previous two. They are characterized by the lowest possible melting point. Each of these groups has different properties that are desirable or not in the design. The best known organic material is paraffin. It is widely used in construction, mainly with gypsum and concrete [14,15,16]. An advantage of paraffin is undoubtedly its ability to mix with other materials. Another important advantage is its heat capacity. Unfortunately, a disadvantage is its flammability. Another fairly well-known PCM is fatty acids. Their advantage is a large temperature range in which the state of aggregation changes from solid to liquid. This means that they can select a substance of interest with a specific phase-change point for a specifically designed case. A disadvantage is their high manufacturing cost. Another group consists of inorganic compounds such as hydrated salts. Such compounds have several useful advantages from the point of view of construction, namely a high coefficient of thermal conductivity which is several times greater compared to paraffin. Another important feature from the point of view of profitability is a low cost of production. Disadvantages include the ability to undercool, making inorganic materials useless in some cases. Eutetic materials are actually a mixture of the previous two materials, making them difficult to predict in terms of heat exchange [6,7,17,18].

One of the key aspects regarding the use of phase-change materials in building materials is the ability to mix them evenly without side effects such as the precipitation of PCM on the surface of the material or, most importantly, the distribution of PCM in the material in such a way that the thermal parameters are the same over the entire surface. In addition to the previously mentioned properties of phase-change materials, it is important that the material is correctly introduced. In this case, microencapsulation is used, i.e., a specially prepared PCM in the form of granules which is resistant to problems with mixing or leakage on the surface of the material. It is caused by the fact that the phase-change material is encapsulated and thus insulated from the external conditions prevailing in the building material [19,20,21,22,23,24]. As such, it is certain that the PCM will not degrade when the phase is changed to a liquid phase. However, a considerable disadvantage of microencapsulation is its expensive production method. In the case of construction, it is important due to the likely high use of it, which translates into the use of phase-change material throughout the entire project.

While analyzing many studies devoted to the study of the influence of microencapsulation on building materials, several observations were presented that may significantly affect the physical properties of the material. One of the phenomena is the change in the properties of the substrate. Sometimes, added PCM reduces the porosity of the material [25]. This has the advantage of increasing mechanical strength by reducing the porosity of the material. Analyzing the research conducted so far, it turns out that a share of more than 20% of phase-change material adversely affects the tested material [26]. This is due to the probable leakage of phase-change material, which can cause pores to form in the dried material when the PCM phase changes. When phase-change material is in the solid phase, it fills the pores in the material; then, when it liquefies, it flows out of the previously filled surface, leaving behind micro-holes. There is another property closely related to porosity. When there are too many pores in the material, the heat transfer process is smaller [25,27]. Based on this theory, the effect of porosity was investigated during a material thawing and freezing test. It was found that the admixture of phase-change material affects the hygroscopicity of the material [28]. As a result of temperature studies, it was found that the share of PCM influences the mass degradation of the entire material, but it also positively influences its resistance to freezing and thawing [29,30]. Other studies confirm the relationship between porosity and the properties of the base material. One study was carried out on a waste polyoxymethylene copolymer additive in order to ascertain how this additive would affect the compressive strength and bending strength of gypsum. On the basis of the obtained results, it was found that an increase in compressive strength and a lack of increase in bending strength were caused by a decrease in the porosity of gypsum samples. This confirms the thesis that the less porous the gypsum is, the higher the compressive strength will be [31].

When studying the effects of phase-change materials on building materials, many scientists have focused on the use of phase-change materials in concrete. However, it is also worth mentioning another material that easily combines with PCM—gypsum. The process of creating plaster for use in construction is quite simple due to its availability.

The most popular solution for using plaster is plasterboard. This is due to its easy and cheap production and its wide applications in construction. The production of such boards is extremely simple because it consists of mixing plaster with various wastes, usually post-production. This is followed by calcination, and the next step is the addition of various substances in order to improve durability, for example. Then, a long line is made, which is cut to the required dimensions. The last stage is the drying of the plates at a temperature above 140 °C.

Referring to the previous information on the wide use of gypsum in construction, this article presents research on a specially prepared mixture that can be an alternative to microencapsulation. In addition, the presented research is a continuation of the previous work [32] which allowed for the isolation of essential ingredients and proportions to make the mixture. In this article, the PCM/gypsum composite was subjected to several mechanical tests, and, importantly, from the point of view of thermal properties, it was possible to investigate the thermal conductivity coefficient in the temperature range wherein phase-change material changes its state to liquid.

The aim of this work was to design, manufacture, and determine the thermal properties of a composite that was based on a mixture of gypsum, paraffin, and polymer. It was decided to determine changes in values for various temperatures of the following parameters of the analyzed material: thermal conductivity, volumetric heat capacity, and diffusivity, as well as the ability to perform tests that might determine the hardening time of the mixture, depending on the content of the PCM and polymer. The assumption of the abovementioned studies is the direct application of the phase-change material, in this case paraffin, to the gypsum mixture. A mixture prepared in this way allows for the preparation of drywall and is thus an alternative to micro-encapsulation, which is expensive to produce. The method of preparation was carried out by dissolving an appropriate amount of polymer in water, and then adding gypsum and paraffin in appropriate amounts. Then, all ingredients were mixed and poured into different molds for the next tests presented in the article.

## 2. Materials and Methods

In order to obtain the most reliable test results for the production of composite samples, raw gypsum (80% pure, Knauf, Rogów, Poland) without additives, and which was prepared for the company Atlas, was sieved on a sieve with a mesh size of 0.5 mm. The properties of gypsum are presented in Table 1.

The PCM we used was RT22HC paraffin manufactured by Rubitherm (Berlin, Germany). The point of transformation from solid to liquid was selected so that it could reflect the conditions inside the room as much as possible, especially when it is necessary to ensure thermal comfort conditions. In order for the paraffin to be correctly incorporated into the mixture, conditions were such that it remained in a liquid state. This made it possible to obtain a homogeneous mixture without the fear of the paraffin quickly changing its physical state upon the addition of water, which would make it impossible to mix thoroughly. The properties of paraffin are shown in Table 2.

On the basis of previous studies [32], the AM 25 polymer was selected. As shown in previous studies, it is a polymer that can be mixed with gypsum and paraffin without a significant deterioration of the sample surface or undesirable phenomena forming, such as cracks, discoloration, or an inhomogeneous structure.

The results of this study confirm the benefits of using a phase-change material as an additive in order to increase the thermal properties of the surface. For this purpose, samples were created and made which had a constant gypsum and paraffin content, as well as a different mass fraction of the polymer. The use of the polymer was to prevent the paraffin from flowing out of the sample (composite) when changing its phase from solid to liquid. The described property of the composite is called paraffin-tight and is an alternative to the microencapsulation process, which is a costly operation for the production of a PCM based on paraffin. In the study, samples with 0.1%, 0.5%, and 1% polymer content were analyzed while, in the heating and cooling tests, the following shares were prepared: 0.1%; 0.2%; 0.4%; 0.6%; 0.8%; 1.0%. Each of the above proportions is a percentage by mass in relation to the mass of the sample.

### 2.1. Heating and Cooling

To exclude the influence of the dimensions of the samples on the obtained results, samples of various shapes were prepared, i.e., 5 cm × 5 cm and 1.5 cm thick which, in the test, were called hexagonal. These samples also corresponded to the thickness of a plasterboard wall and are cylindrical with a diameter of 5, 5 cm and a height of 5.5 cm. For each of the mentioned samples, the proportion of polymer amounted to 0.1%; 0.2%; 0.4%; 0.6%; 0.8%; 1.0%. Additionally, 12 samples were made according to the following recipe:

1. An aqueous polymer solution containing 0.1% was formed with 0.2%, 0.4%, 0.6%, 0.8%, and 1.0%, and then mixed;

2. The prepared solution was combined with gypsum in a mass ratio of 0.65:0.35;

3. Paraffin was added to the resulting mixture in a mass ratio of 0.2 paraffin: 0.8 of the mixture;

4. It was mixed with a magnetic stirrer for 15 s until a homogeneous mixture was obtained.

### 2.2. Tests of Hardening Time of Individual Samples—Vicat Apparatus

The test of the hardening time of the mixture (a basic test determining the quality of building materials) was carried out with the use of a manual Vicat apparatus.

In each case, hardening tests were carried out in triplicate for each type of sample. Twelve measurements were taken: 3 for raw gypsum, 3 for the sample with 0.1% polymer, 3 for 0.5% polymer, and 3 for 1% polymer. The test consisted of preparing a cylindrical form into which the mixture was poured, and then the time that it took from pouring it to the moment the apparatus pin was immersed in the analyzed mixture to a depth of 6 mm was measured. The study was then considered complete. The plunger dipping time was measured in minutes.

### 2.3. Testing the “Ability” of the Material to Accumulate Paraffin—An Uptake Test

In order to confirm the thesis that the use of polymer in gypsum allows the paraffin to be retained in the center of the composite, an “uptake” test was used. In the tests performed, paraffin was used instead of water. In order to obtain the most reliable results in the research, 20 cylindrical samples were used. The dimensions of the samples were the same as for the previously performed drying and cooling tests for cylindrical samples, and they were prepared according to an identical procedure. The samples differed in the percentage of polymer content, so the tests used gypsum with a polymer content of 0%, 0.1%, 0.5%, and 1.0%. For each proportion of polymer in the gypsum, 5 samples were prepared, which allowed us to obtain the final result as the arithmetic mean of the conducted experiments. The samples were weighed every minute for the first 10 min. Subsequent measurements were made every 5 min. Total measurement time was 30 min.

### 2.4. Thermal Conductivity, Volumetric Heat Capacity, and Diffusivity

Another tested parameter describing the thermal properties of the composite was the determination of the thermal conductivity coefficient. It is one of the most important thermal parameters which characterize individual building materials, especially insulation materials. The test was performed using the Isomet 2114 device(Applied Precision Ltd., Bratislava, Slovakia), which is equipped with a needle measuring probe. In order to take into account the influence of temperature changes on the value of the thermal conductivity coefficient λ, the samples were placed in a special cabinet that stabilizes thermal conditions. The measurement was performed in the range of temperature changes between 10 °C and 35 °C. The samples were conditioned every 1 °C, 10 measurements were made in each experiment, and the final result was determined as the arithmetic mean of the measurements. The dimensions of the samples were set at 40 mm × 40 mm × 160 mm. The proportion of polymer used was the same as previously mentioned, namely, 0.1%, 0.5%, and 1%. The volumetric heat capacity and thermal diffusivity of the material were also measured with the Isomet device (Table 3).

## 3. Results

### 3.1. Heating and Cooling

The first test to see whether the polymer actually changes the properties of the gypsum enough to improve the impermeability of the paraffin is the heating and cooling cycle. The idea behind this study was to simulate the change in outside temperature in order to approximate the behavior of the sample under real conditions. This is important for the use in construction, as most building materials are exposed to weather conditions and particular temperatures. Samples used for the tests had 0.1%, 0.2%, 0.4%, 0.6%, 0.8%, and 1% polymer content. The tests were carried out for two shapes of sample (cylindrical and hexagonal). In the figures below, the mass statistic mainly relates to the loss of paraffin during phase transitions.

The test was carried out at two temperatures: 8 °C and 40 °C. To simulate paraffin phase-change conditions, the temperature was changed every day from 8 °C to 40 °C, and on the second day from 40 °C to 8 °C. The choice of the temperature range was suggested by the transformation point of the paraffin we used, which changes the aggregate state at 22 °C. Each day, the samples were weighed to determine their mass change gradient. Each sample was evaluated separately for different starting weights.

The relative mass change of the tested samples was determined from the dependence (1):

(1) $$M_{l}=\frac{M_{n}-M_{n-1}}{M_{n}}\;\cdot 100\%,$$

where *M_n_* = the mass of the sample in a given cooling/drying state [g] and *M_n−_*_1_ = the previous sample weight [g].

**Figure 1:**
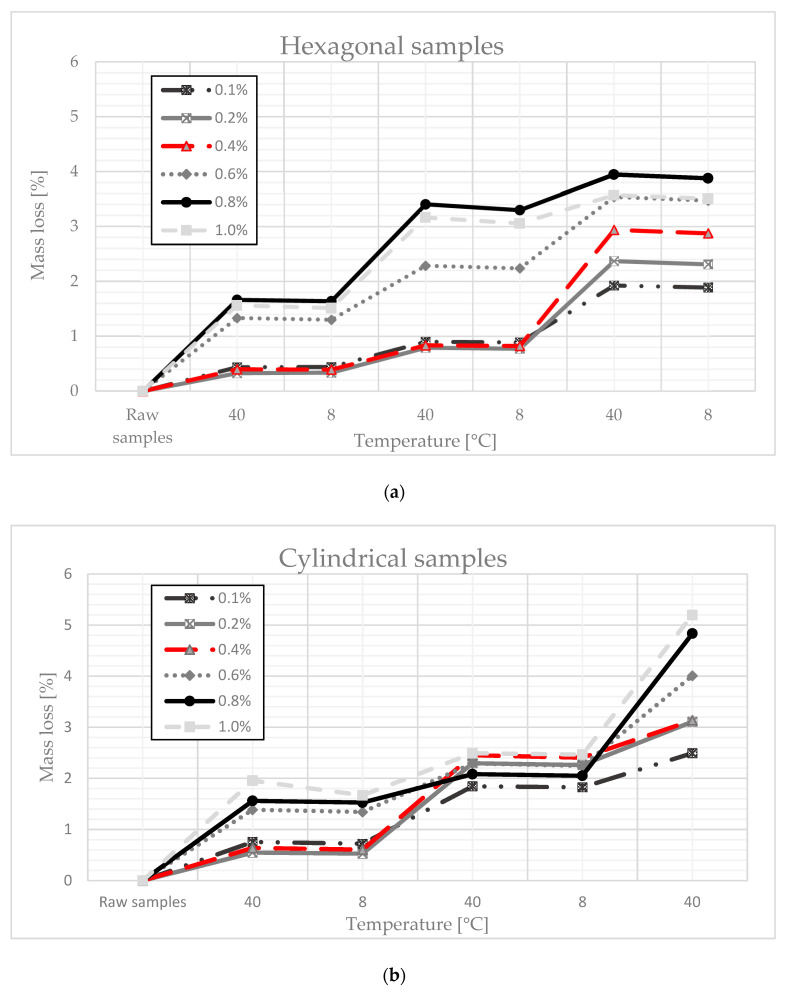
(**a**) Mass loss due to changing temperature conditions (hexagonal samples). (**b**) Mass loss due to changing temperature conditions (cylindrical samples).

The results presented in Figure 1a,b confirm the influence of the polymer content on the weight loss of the sample subjected to cyclic drying and cooling. Comparing the graphical relationships presented in Figure 1a,b, it can also be seen that the weight loss depends on the shape of the sample while, for the same polymer shares, cylindrical samples have a greater weight loss than the cube-shaped samples. This is related to the dimensions of the analyzed samples and their external surface. In the case of cylindrical samples, weight loss was about 5% (with a polymer content of 1%). Hexagonal samples, the dimensions of which simulated the thickness of the plasterboard, showed smaller relative weight losses due to evaporation or paraffin leakage at higher temperatures (in the worst case, weight loss was 4% with 0.8% polymer content). The polymer content of 0.1% caused a loss of approx. 2%, which is the best result that allows for further testing. That said, the best result for the cylindrical samples, which was 2.5% for a polymer content of 0.1%, shows that the material thickness slightly affects the outflow of paraffin from the surface. The test was completed after five cycles of temperature change. This was due to the fact that, in the next cycle, the weight loss was insignificant, which means that there was a situation in which the mass loss stopped.

### 3.2. Tests of Hardening Time of Individual Samples—Vicat Apparatus

This paper presents the results of research on the setting time of a mixture, which is one of the basic parameters for determining the suitability of building materials in the form of a mortar. During the experiments, a Vicat apparatus with manual operation (manual according to PN-EN 196-3 MMC-0051/E, Multiserw, Marcyporęba, Poland) was used. The results of testing the hardening time of gypsum composite samples with a polymer content of 0–1% are presented in Table 4. The results presented therein are the arithmetic mean of three independent measurements for each analyzed proportion of polymer.

On the basis of the obtained results, it can be seen that the hardening time for the sample with 0.1% polymer content is shorter than the hardening time for the sample without the polymer (raw gypsum), which should be considered favorable. Our ratio of 0.65:0.35 for water to gypsum is approximately similar to the gypsum hardening results in other works. In [33], gypsum was presented in relation to how a water level of 0.66 reached the sinking of the needle in about 21 min. In this study, the raw plaster achieved this result in 21 min and 28 s. In [33], tests were also carried out on gypsum mixed with cellulose ether where, like the polymer, it extended the setting time depending on the degree of admixture. A long hardening (binding) time of samples with 0.5% and 1%, thus exceeding 30 min, excludes these samples from further analysis and potential use.

### 3.3. Testing the “Ability” of the Material to Accumulate Paraffin—An Uptake Test

**Figure 2:**
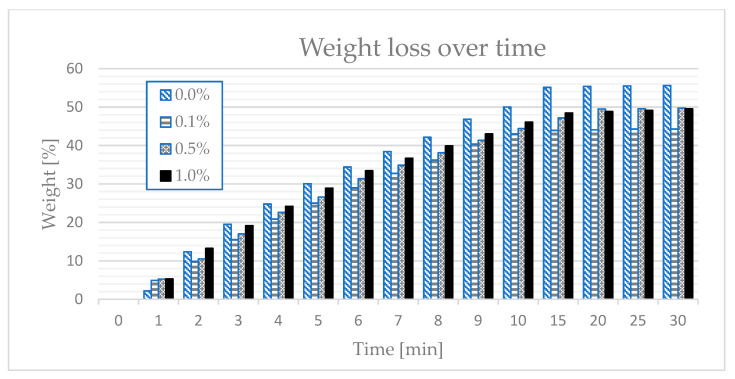
Results of the paraffin rising test.

This paper proposes a modified method for determining the ability of a composite to store paraffin—the “uptake test”. In the discussed case, paraffin was used instead of water for the classic uptake test. Samples for the uptake test were made as similar to those used in the drying and cooling tests, meaning they had a cylindrical shape. The measurement was performed within 30 min for each tested proportion of polymer in the sample. In order to obtain a reliable test result, five samples were used, and the final result was the arithmetic mean of five obtained results. As a function of time for different polymer concentrations in the sample, the values of the obtained arithmetic means show the graphical relationships presented in Figure 2.

As can be seen in Figure 2, in the case of the sample without the polymer, the mass of paraffin accumulated in the composite quite quickly reached 50% of the total mass. This means that there was no “blockage” to stop the paraffin from rising. The situation was different in the case of samples with a polymer content of 0.1%. It can be seen that, for 15 min, the paraffin mass was about 44% of the initial mass.

A downward trend could also be seen in the samples with 0.5% polymer content, in which paraffin was 45% of the initial weight. Additionally, less pull-up tendency was observed in samples containing 1% polymer. This means that the presence of the polymer has a positive effect on the retention of paraffin inside the sample; although, on the basis of these tests, it can be concluded that the optimal result was obtained for samples with a polymer content of 0.1%.

### 3.4. Thermal Conductivity, Volumetric Heat Capacity, and Diffusivity

The final test we implemented was performed to determine the value of the thermal conductivity coefficient. This is important because the value of this factor is used to determine the suitability of individual materials for utilitarian applications. The performed test was enriched by conditioning the measurement with a different temperature in the range of 10–35 °C. Measurements were made every 1 °C. Each time, 10 measurements were made, and the final result was determined as the arithmetic mean of these measurements (Table 5).

**Figure 3:**
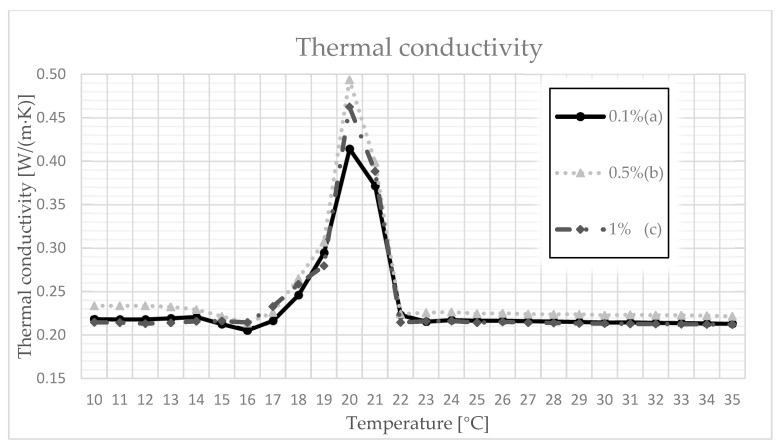
Thermal conductivity as a function of temperature for (a) 0.1%; (b) 0.5%; (c) 1% polymer content.

As can be seen in Figure 3, the thermal conductivity coefficient changes with an increase in temperature. The greatest change can be seen in the temperature range 18–22 °C, where a clear peak for each of the samples is in the range of 0.4–0.5 W/(m·K). This means that the paraffin inside the samples began to change its aggregate state under the influence of temperature. For the values of 0.5% and 1%, the coefficient reached a value close to 0.5 W/(m·K). It is also interesting that, after exceeding 22 °C, the coefficient was set at the level before the phase change.

**Figure 4:**
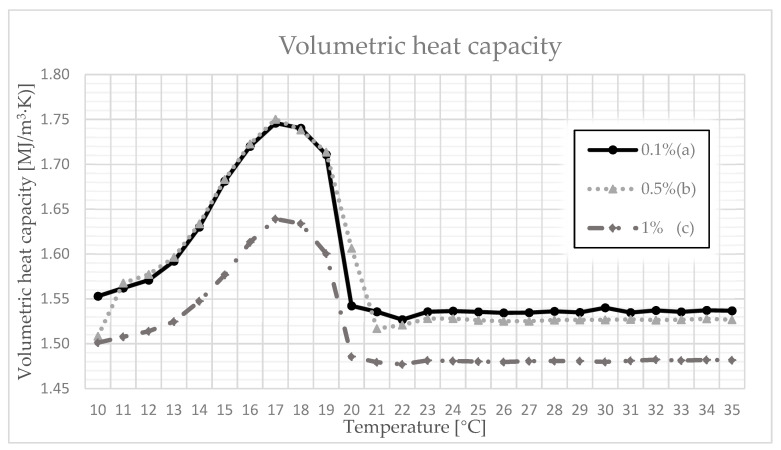
Volumetric heat capacity as a function of temperature for (a) 0.1% polymer; (b) 0.5% polymer; and (c) 1% polymer.

As can be seen in Figure 4, volumetric heat capacity changes its value as a function of temperature. Observing the dependencies in Figure 4, it can be observed that the heat capacity systematically increases with an increase in temperature, at least to the limit defined by a temperature of 17 °C. Then, it decreases and, at a temperature of 20 °C, it reaches a value close to the initial value and stabilizes, despite further temperature increases. By analyzing the dependencies in Figure 4, it can be observed that the composites with 0.1% and 0.5% polymer content at 17 °C are characterized by a volumetric heat capacity value of 1.75 MJ/(m^3^·K), and the sample with 1% polymer content reaches a maximum of 1.64 MJ/(m^3^·K). The obtained relations prove the influence of the addition of paraffin on the value of the conductivity coefficient, as well as on the volumetric heat capacity of the designed composite.

**Figure 5:**
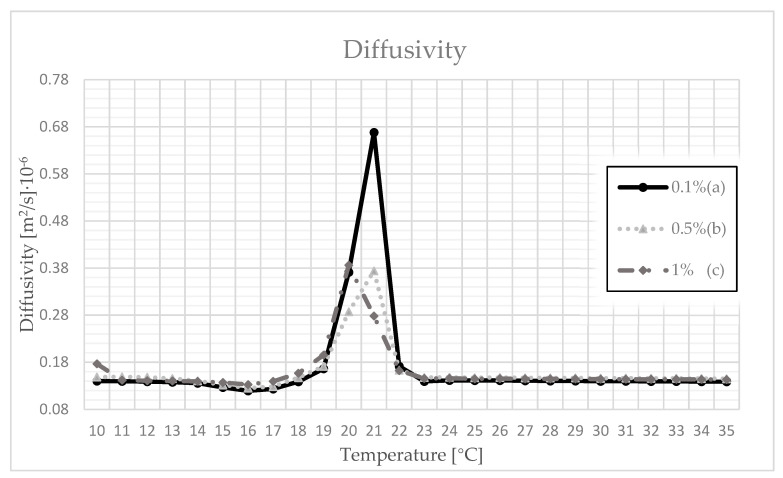
Changes in the value of thermal diffusivity as a function of temperature for different percentages of polymer in the composites with (a) 0.1% polymer; (b) 0.5% polymer; (c) 1% polymer.

As can be seen in Figure 5, thermal diffusivity, a parameter that depends on the two previously measured values, alters its value depending on temperature changes. In a similar fashion to the previous indicators, the greatest increase in its value can be noticed for temperatures close to the phase transition temperature of the used paraffin. The maximum value for samples with a polymer concentration of 0.1% or 0.5% was obtained at a temperature of 21 °C while, for a concentration of 1%, the maximum value was obtained at 20 °C. The highest value of thermal diffusivity, approx. 0.7·10^−6^ m^2^/s, can be observed for the sample with 0.1% polymer content.

## 4. Conclusions

Based on the conducted research, several conclusions can be drawn:

- When the composite was subjected to a drying and cooling cycle, some mass of paraffin leaked from the composite. The loss of paraffin mass depended on the shape and mass of the sample. For cylindrical samples, the weight loss was 5% and, for cubic samples, the maximum weight loss was 4%, which should be considered a satisfactory result;
- The measurement of the curing time value showed that the polymer additive used in the composite influenced the hardening time of the samples. The PCM sample with the addition of 0.1% polymer achieved a hardening time that was shorter than that of the raw gypsum;
- A modified, proprietary study of paraffin rising proved the benefits of using the polymer in the designed composite. The sample with the addition of polymer soaked slower than the sample made of gypsum alone;
- The thermal conductivity coefficient increased to its maximum value, which was characterized by the phase transition point of the paraffin used, and then adjusted itself to the initial value. It can be assumed that, during the transformation, the designed composite can absorb so much heat that, after another change in aggregate state, it can release the stored heat, e.g., at night, which is a chief idea behind the use of PCM.

## Figures and Tables

**Table 1:** Properties of gypsum used for research.

Parameter	Unit	Result
Calcium sulfate hemihydrate content	%	>95
(β-CaSO_4_·0.5H_2_O) Crystallization water	%	5.6–6.0
Mechanical strength after drying to constant weight - for bending - for compression	MPa	>5.0 >12.0

**Table 2:** Properties of parafin RT22HC used for research.

Parameter	Unit	Result
Melting area	°C	20–23
Congealing area	°C	23–20
Heat storage capacity	kJ/kg	190
Specific heat capacity	kJ/kg·K	2
Density solid	kg/L	0.76
Density liquid	kg/L	0.7

**Table 3:** Laboratory equipment used to perform the tests.

Type of Equipment	Parameter	Model and Company	Country, City
Dryer	Temperature	Model ST3 company Pol-eko	Poland, Wodzisław Śląski
Device for testing the thermal conductivity	Thermal conductivity coefficient, volumetric heat capacity, thermal diffusivity	Isomet 2114 company Applied Precision Ltd.	Slovakia, Bratislava
Vicat apparatus	Drying time	manual according to PN-EN 196-3 MMC-0051/E by Multi-serv	Poland, Marcyporęba

**Table 4:** Results of drying time of individual samples.

Percentage of Polymer	Drying Time
0%	21 min 28 s
0.1%	19 min
0.5%	38 min 42 s
1%	55 min 30 s

**Table 5:** Results obtained during measurements of gypsum thermal coefficients.

Percentage of Polymer	Temperature	Thermal Conductivity			Volumetric Heat Capacity			Diffusivity		
	°C	W/m·K			MJ/(m^3^·K)			(m^2^/s) 10^−6^		
%		Min	Max	Average	Min	Max	Average	Min	Max	Average
0.1	17	0.210	0.219	0.217	1.727	1.755	1.746	0.118	0.125	0.123
	18	0.215	0.249	0.246	1.723	1.755	1.740	0.123	0.143	0.139
	19	0.246	0.302	0.295	1.703	1.722	1.711	0.143	0.178	0.166
	20	0.304	0.546	0.414	1.518	1.695	1.542	0.256	0.514	0.371
	21	0.345	0.403	0.372	1.521	1.544	1.536	0.443	0.828	0.668
	22	0.209	0.250	0.223	1.520	1.534	1.527	0.137	0.222	0.171
0.5	17	0.214	0.227	0.226	1.727	1.767	1.750	0.124	0.130	0.128
	18	0.225	0.269	0.265	1.721	1.751	1.738	0.129	0.156	0.147
	19	0.267	0.315	0.307	1.703	1.732	1.713	0.154	0.185	0.171
	20	0.454	0.548	0.494	1.551	1.657	1.606	0.204	0.353	0.287
	21	0.334	0.470	0.399	1.510	1.528	1.517	0.221	0.489	0.374
	22	0.218	0.241	0.224	1.515	1.528	1.521	0.143	0.215	0.163
1	17	0.221	0.250	0.233	1.629	1.655	1.639	0.135	0.152	0.139
	18	0.247	0.272	0.258	1.626	1.647	1.634	0.151	0.166	0.157
	19	0.269	0.298	0.280	1.572	1.621	1.600	0.166	0.229	0.195
	20	0.431	0.510	0.462	1.470	1.499	1.486	0.292	0.489	0.386
	21	0.339	0.480	0.388	1.469	1.480	1.479	0.229	0.325	0.278
	22	0.208	0.224	0.214	1.473	1.482	1.477	0.141	0.181	0.162

## Data Availability

Data are contained within the article.
